# Supplementary figures and images for: Diversity and ice nucleation activity of Pseudomonas syringae in drone-based water samples from eight lakes in Austria
Source: PeerJ. 2023 Nov 28;11:e16390. doi: 10.7717/peerj.16390 (PMC10691352; doi:10.7717/peerj.16390)

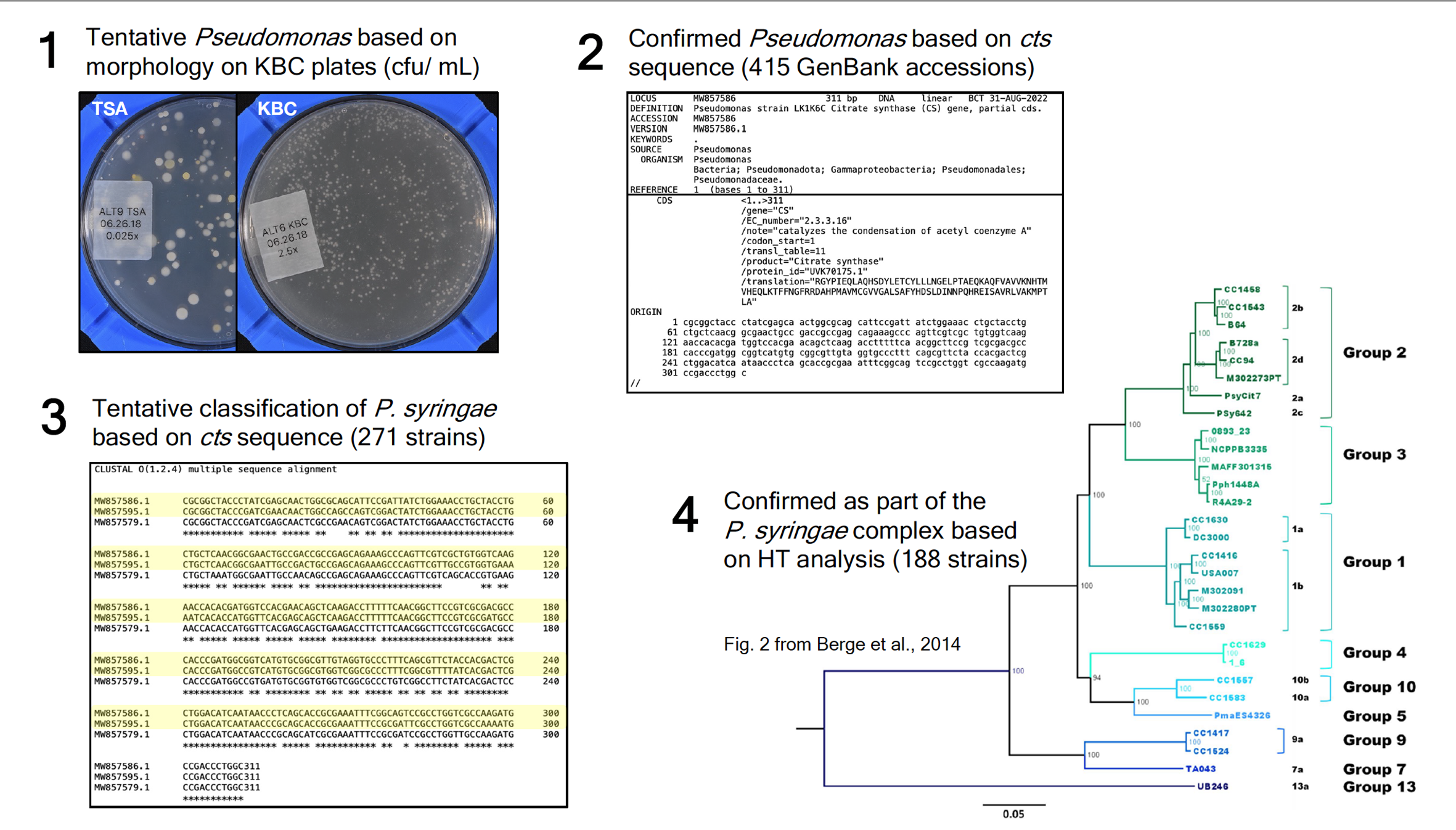

Supplement: Supplemental Information 2 — (1) Bacterial growth shown for a TSA and KBC plate from Lake 1 (ALT). Colonies from KBC became the set of tentative Pseudomonas stains. (2) GenBank accession of MW857586 from the set of 415 confirmed Pseudomonas strains that were verified by partial sequencing of the cts gene. (3) Example of a sequence alignment of tentative P. syringae strains. The highlighted strains, MW857586.1 and MW857595.1, grouped with P. syringae, while MW857579.1 did not. (4) Visual of phylogeny example of P. syringae strains mapping to different phylogroups in the P. syringae complex. [file peerj-11-16390-s002.png]
