# Supplementary material for: Diversity and ice nucleation activity of Pseudomonas syringae in drone-based water samples from eight lakes in Austria
Source: PeerJ. 2023 Nov 28;11:e16390. doi: 10.7717/peerj.16390 (PMC10691352; doi:10.7717/peerj.16390)
Supplement: Supplemental Information 3 — Lake name abbreviation, collection date, number of tentative P. syringae strains from each lake, number of tentative P. syringae strains assayed for ice+ (251 of 271), total number of tentative P. syringae ice+ frozen samples, percent of frozen ice+ strains from tentative P. syringae. [file peerj-11-16390-s003.docx]

|  |  |  |  | | | |  |  | |  | | |  |
| --- | --- | --- | --- | --- | --- | --- | --- | --- | --- | --- | --- | --- | --- |
| **Abbreviation** | **Date of Collection** |  | **Tentative classification of**  ***P.syringae* based on *cts* sequences** | **Strains Tested for ice+** |  | **Total Ice+** | | | **Percent Ice+** | |  |  |  |
| ALT | June 7 2018 |  | 26 | 26 |  | 3 | | | 12% | |  |  |  |
| GRU | June 7 2018 |  | 53 | 50 |  | 19 | | | 38% | |  |  |  |
| TOP | June 7 2018 |  | 53 | 44 |  | 8 | | | 18% | |  |  |  |
| GOS | June 8 2018 |  | 41 | 40 |  | 14 | | | 35% | |  |  |  |
| GOL | June 8 2018 |  | 24 | 23 |  | 6 | | | 19% | |  |  |  |
| HIN | June 8 2018 |  | 21 | 20 |  | 13 | | | 65% | |  |  |  |
| OSS | June 10 2018 |  | 49 | 44 |  | 13 | | | 30% | |  |  |  |
| WOR | June 10 2018 |  | 4 | 4 |  | 0 | | | 0% | |  |  |  |
| All Lakes | Total |  | 271 | 251 | 76 | |  |  |  |  |  |  |  |
